# Understanding the Intricate Web of Phytohormone Signalling in Modulating Root System Architecture

**DOI:** 10.3390/ijms22115508

**Published:** 2021-05-24

**Authors:** Manvi Sharma, Dhriti Singh, Harshita B. Saksena, Mohan Sharma, Archna Tiwari, Prakhar Awasthi, Halidev Krishna Botta, Brihaspati Narayan Shukla, Ashverya Laxmi

**Affiliations:** National Institute of Plant Genome Research, Aruna Asaf Ali Marg, New Delhi 110067, India; manvi.sharma3@yahoo.com (M.S.); dhritisingh89@gmail.com (D.S.); harshita@nipgr.ac.in (H.B.S.); sharma.mohan642@gmail.com (M.S.); archnatiwari@nipgr.ac.in (A.T.); prakhar@nipgr.ac.in (P.A.); bhkrishna@nipgr.ac.in (H.K.B.); bn.shukla07@nipgr.ac.in (B.N.S.)

**Keywords:** root system architecture, root system plasticity, root development, root tropic responses, root meristem, phytohormone signalling

## Abstract

Root system architecture (RSA) is an important developmental and agronomic trait that is regulated by various physical factors such as nutrients, water, microbes, gravity, and soil compaction as well as hormone-mediated pathways. Phytohormones act as internal mediators between soil and RSA to influence various events of root development, starting from organogenesis to the formation of higher order lateral roots (LRs) through diverse mechanisms. Apart from interaction with the external cues, root development also relies on the complex web of interaction among phytohormones to exhibit synergistic or antagonistic effects to improve crop performance. However, there are considerable gaps in understanding the interaction of these hormonal networks during various aspects of root development. In this review, we elucidate the role of different hormones to modulate a common phenotypic output, such as RSA in Arabidopsis and crop plants, and discuss future perspectives to channel vast information on root development to modulate RSA components.

## 1. Introduction

Plants are undeniably the most important source of food, fuel, medicines, and fibres. Apart from the above ground parts of the plants that are actively involved in photosynthesis, the hidden half i.e., the root, controls a wide variety of processes such as nutrients and water acquisition and anchorage in the soil, and also acts as an interface between plant and the earth atmosphere. By sensing and responding to environmental cues and soil nutrition conditions, roots are capable of dramatically altering their architecture and hence enable plants to overcome the challenges posed due to their sessile nature.

Understanding the development and architecture of roots holds potential in improving crop yield and optimizing agricultural land use. The recent development of newer technologies such as advanced digital photography, 3D root imaging, transparent soils, automated rhizotron, X-ray computed tomography, luminescence-fluorescence based imaging systems, and neutron tomography has enabled us to better understand the architecture of complex root systems [1]. Moreover, extensive studies on the Arabidopsis root system have generated crucial insights on molecular mechanisms regulating root architecture development. RSA is influenced by the cross-talk of different hormones as well as by hormone-environment factors that integrate with the plant system via a specific set of downstream regulators that lead to changes in gene expression, signal transduction, and metabolic conversions [2,3]. With the advent of recent technology, the identification of crucial downstream regulators and signalling pathways has now become a reality. For instance, a study by Šimásková and co-workers has shown that cytokinin (CK) induces PIN1 and PIN7 expression via CYTOKININ RESPONSE FACTOR 2 (CRF2) and CRF6 in order to modulate RSA. They showed that domains PCRE7 and PCRE1 of PIN1 and PIN7, respectively, were responsible for their cytokinin-inducibility. A yeast one hybrid (Y1H) screen, ChIP-qPCR, and a protoplast-based luciferase assay demonstrated that the CRF2 and CRF6 transcription factors are positive regulators of PIN7 and PIN1 expression. Additionally, mutant analysis showed that *crf236* phenocopies auxin transport defective mutants, confirming that PINs are targets of these transcription factors [4]. Another study shows that FOUR LIPS (FLP) is a transcriptional regulator of PIN3 in the early stages of LR development [5]. However, such interactions have mainly been characterized in model plants such as Arabidopsis, and translating this information into economically important crop plants will open new research possibilities for plant biologists.

This review will briefly outline the root development process from the formation of an embryonic root to the control of root architecture. It also provides insights on various aspects of RSA and how they are regulated by various hormones and their crosstalk in plants.

## 2. Root System Architecture

**Figure 1:**
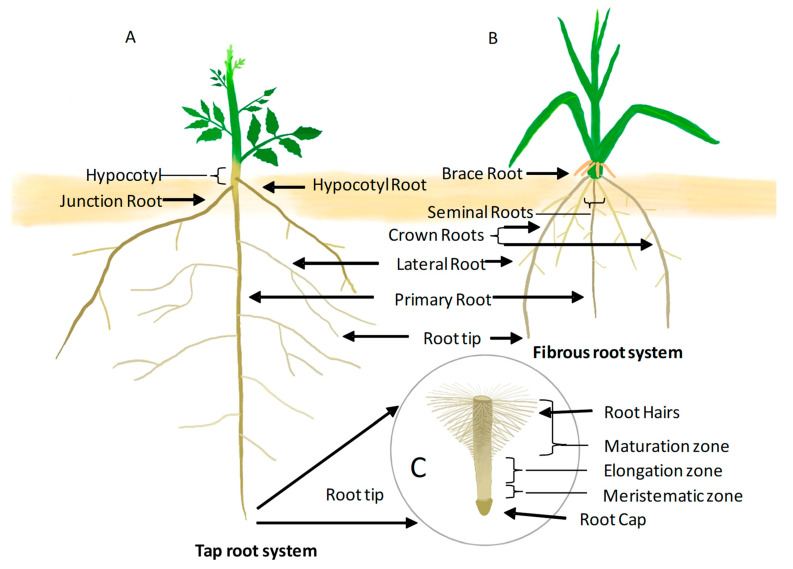
(**A**) Tap root system: RSA of a dicot (tomato) showing embryonic primary root, lateral roots, adventitious roots of shoot origin (hypocotyl root), and root-shoot junction (junction root). (**B**) Fibrous root system: RSA of a monocot (maize) showing embryonic origin primary root and seminal roots, adventitious roots of shoot origin (brace roots) and root-shoot junction (crown root), laterals, and root tip. (**C**) Root tip of primary root showing root cap, meristematic zone, elongation zone, and maturation zone having root hairs.

The three dimensional structure of the root system is specified as root system architecture (RSA). RSA components include the primary root (PR) length, the number, angle, length, patterning of lateral roots (LR), adventitious roots (AR), and epidermal cell outgrowth called root hair (RH) [6]. There are two types of root systems defined by their branching patterns and developmental origin: taproots and fibrous roots (Figure 1). Taproots that occur in dicots consist of the PR, RH and secondary smaller LRs, and ARs (arising from a root-shoot junction or from the hypocotyl) (Figure 1A). In addition to RH and primary root, a fibrous root system as observed in cereals (*Oryza sativa*, *Zea mays* etc.) consists of dense mass of adventitious roots (AR) that occur along with seminal roots, crown roots, and LRs (Figure 1B). However, in cereals, the PR dies as the monocots age, and the adventitious roots form the major portion of the RSA. Depending on the species, RSA displays high variation in morphological traits that help the plant to adapt to a highly competitive environment.

### 2.1. Primary Root Growth and Development

PR is the basic and main component of RSA, is the first to emerge, and is derived from embryonically formed meristematic tissue that forms the hypophysis near the base of the early globular embryo. Asymmetric division takes place, giving rise to upper cell that eventually forms the quiescent centre (QC) and the lower cell that gives rise to the upper cells of the columella. Numerous stem cells surround the QC and, depending on their position, give rise to different tissue types such as columella, vascular tissue, ground tissue, epidermis, and the root cap [7].

The primary root has different developmental zones. In the root apical meristem (RAM), all cells originate from one or more precursor or stem cells at the very tip, called stem cell niche (SCN). The daughter cells divide at different proportions and various rates to generate the division zone (DZ). These cells exit the cell cycle and elongate, generating the elongation zone (EZ). The cells then differentiate and acquire tissue specific features based on their radial position. This zone is called the differentiation zone (Diff zone). The transition zone (TZ) marks the developmental boundary between dividing and differentiating cells [7,8]. Each developmental zone correlates with the graded distribution of hormones, mRNA, peptides, TFs, etc. Auxin and CK play an important role in maintaining the SCN and cell differentiation, respectively. Auxin gradient is expressed as a maximum in SCN and shows a decline towards the TZ [9,10]. The expression of PLETHORA (PLT) TFs correlates with the auxin maxima, declining at the TZ [11], and its expression in SCN is dependent on ARFs such as MONOPTEROS, NON-PHOTOTROPHIC HYPOCOTYL 4 (NPH4) [12]. In turn, PLT contributes to the establishment of auxin maxima by increasing auxin biosynthesis and polar auxin transport (PAT). Cell expansion and differentiation in the root are controlled by CK signalling that promotes the transcription of SHY2 and GH3.17 genes that further shape the auxin gradient, where they position auxin minima at the TZ, leading to differentiation. CKs via ARR1 also promote cell wall remodelling enzyme EXPANSIN1 (EXP1), consequently driving cell differentiation [8].

There are various TFs and genes involved in QC and RAM maintenance. These include *WUSCHEL-LIKE HOMEOBOX 5* (*WOX5*), *CYCLIN D3* (*CYCD3*), *CYCLING DOF FACTOR 4* (*CDF4*), SCARECROW (SCR), SHORTROOT (SHR), HAIRY MERISTEM (HAM), etc. (extensively reviewed in [13]).

There are some substantial differences between dicot and monocot root tissue organization. The cereal tissues are overall larger and more complex with wide cortical cell layers, as compared to the narrow ones in dicots. Additionally, the QC population is drastically larger compared to dicots such as Arabidopsis. Monocots possess polyarch xylem as compared to the tetrarch xylem of dicots. Further, the number of xylems and phloems in dicots vary from 2–8 compared to more than 8 in monocots. In dicots, the pith region is either absent or small and underdeveloped; whereas in monocots the pith is larger and well-developed [14].

### 2.2. Lateral Root Development

LRs provide better anchorage in soil, help in nutrient assimilation and water uptake, and make symbiotic association with microbes. LR development is primarily governed by auxin activity and auxin response maxima; the downstream signalling cascade regulates LR patterning in angiosperms [15,16,17]. LR formation occurs in the single-layered pericycle tissue at the xylem poles, which are termed as xylem pole pericycle (XPP) cells (extensively reviewed in [15]). After continuous anticlinal division and cell elongation, XPP cells move from meristem to a zone of auxin maximum. However, this auxin maxima is not regular; rather, it appears to behave in an oscillation manner, as high auxin peaks are interrupted by low auxin response [18]. The occurrence of the auxin maxima can be seen by DR5:GUS and DR5:luciferase reporters and can be correlated with LR initiation. In monocots, LR initiation differs, as LRs emerge from phloem poles rather than from XPP. In addition, there can be numerous phloem poles (usually 10 or more); LRs initiate in longitudinal files, with the number of files roughly proportional to the stele diameter [19]. Similar to Arabidopsis, the formation of auxin response maxima is important in LR initiation in cereals such as maize. DR5::RFP lines have indicated the presence of auxin response maxima in differentiating xylem cells and in cells surrounding the protophloem vessels [19]. Additionally, genome-wide transcriptome analysis of LR initiation has highlighted the presence of a common transcriptional regulatory pathway between Arabidopsis and maize, suggesting that genes involved in LR initiation are conserved across angiosperms [20].

### 2.3. Adventitious Root Development

Postembryonic roots arising from tissues other than roots are termed adventitious. These may be crown roots (from nodes below the ground), brace root (from nodes above the ground), stem roots, or seminal roots [21]. Different root types contribute to structural and absorbance functions. Primary and seminal roots are mostly important in the first stages of plant growth. Brace roots, which take over at later stages of the monocot RSA, play an important role in the structural support of the plants [22].

### 2.4. Root Hair Development

RH are tip growing extensions from the root epidermis that not only increase the root surface area for better nutrient and water acquisition but also facilitate adhesion of the root to the surrounding rhizosphere and interaction of the root with soil microorganisms such as arbuscular mycorrhizal fungi and nitrogen-fixing bacteria. Several reports cite that overall plant fitness is compromised in several loss-of-function RH mutants when grown in challenging soil conditions [23,24]. Hence, dynamic RH morphogenesis is a trait that can be exploited agriculturally to improve water and nutrient acquisition in heterogeneous soil conditions. RH development starts when a position-dependent signal originates from the underlying cortical cells. Signal perception in epidermal cells that overlie two cortical cells triggers a transcription factor cascade that ultimately inhibits the expression of *GLABRA2* (*GL2*), a core non-RH-determining transcription factor. This causes the expression of *ROOT HAIR DEFECTIVE 6* (*RHD6*) and its homolog *RHD6-LIKE1* (*RSL1*). These proteins induce the expression of downstream basic helix-loop-helix (bHLH) family targets *RSL2*, *RSL4* and homolog of *Lotus japonica ROOTHAIRLESS1* (*LjRHL1*), *LRL3* for root hair growth [25]; among them, RSL4 is the key regulator (extensively reviewed in [26,27,28]). Several hormonal cues participate in the development and regulation of RH, and their crosstalk is important to dynamically regulate RH function in changing soil conditions [27].

## 3. Impact of Hormones in the Regulation of Different Aspects of RSA

### 3.1. Primary Root

The long standing link between auxin and root development dates back to the time of its discovery, when it was named as root forming hormone [29], and is indispensable in regulating almost every major phase of root growth and development [11,17,30,31]. Low concentrations of auxin have been shown to stimulate PR growth in Arabidopsis and maize, while higher concentrations inhibited root growth through auxin receptor TRANSPORT INHIBITOR RESPONSE 1 (TIR1) mediated signalling via an unknown non-transcriptional mechanism [32,33]. It can be speculated that free Aux/IAA proteins might promote root growth, and ubiquitinated Aux/IAA proteins may lead to inhibition of root growth. Among other signals, auxin is a key instructor of root meristem organogenesis on which multiple phytohormones and other signalling pathways converge to regulate root development. In QC, polar auxin transport (PAT) is an important prerequisite to establish auxin response maxima; however, localized auxin production in the roots also contributes to the establishment of auxin gradient and maxima required for normal root development [34,35]. It was assumed that shoot-derived auxin and its downward transport contributes to a major portion of auxin in the root to create auxin maxima. However, recent reports suggest that auxin is produced locally in the roots via a tryptophan-dependent biosynthesis pathway, which converts tryptophan to indole-3-pyruvic acid (IPyA) to indole-3-acetic acid (auxin) [36,37]. WEAK ETHYLENE INSENSITIVE 8 (WEI8) and its homolog protein TRYPTOPHAN AMINOTRANSFERASE RELATED 2 (TAR2) are the key enzymes that produce and maintain auxin homeostasis in the roots, thereby establishing the root meristem. Plants mutated in *wei8tar2* displayed loss of root meristem and reduced root auxin activity. Additionally, through a combination of grafting experiments, Brumos et al. 2018 showed that shoot-derived auxin could not rescue deficiencies of auxin production in roots [36]. Another tryptophan-dependent auxin biosynthesis pathway, which contributes to a lower but significant amount of auxin in roots, was also reported where the root-derived auxin biosynthesis was abolished in *cyp79B2cyp79B3*, thus suggesting multiple sources of auxin production in roots [34]. Localized auxin production and response machinery along with PAT work together to develop flux separation to maintain auxin maxima in QC [38]. There is also a feedback mechanism of WOX5 and INDOLE-3-ACETIC ACID INDUCIBLE 17 (IAA17) in maintaining auxin maximal response in QC cells that regulate the root patterning in distal stem cell niche. WOX5 activity induces auxin production in QC; however, auxin negatively regulates the *WOX5* transcription [39]. IAA17 causes the inhibition of auxin response and therefore promotes *WOX5* transcription. IAA17-dependent auxin response suppression restricts the *WOX5* expression in QC and therefore establishes the identity of root stem cells. Gain-of-function mutant of *IAA17*(*axr3-1*) displayed enhanced auxin maxima in QC and reduced auxin response in distal stem cells, leading to the inhibition of root distal stem cell differentiation [38].

In a recent report, a rice gene named OsFPFL4 modulates root growth by affecting auxin levels. OsFPFL4 overexpressing plants showed shorter primary roots, which were due to more auxin accumulation and altered expression of auxin biosynthesis and transport-related genes [40]. Another gene encodes for NITRILASE 1 enzyme in Arabidopsis, which regulates root growth by modulation of auxin biosynthesis. NIT1 overexpressing plants showed shorter primary roots, which were due to drastic changes of both free IAA and IAN levels [41]. In rice, OsMADS25 regulates root growth through an increased accumulation of auxin in the root. OsMADS25 promoted auxin biosynthesis as well as transport. At the same time, it also reduced auxin degradation to stimulate root growth [42]. IAA also induces cyclic guanosine 3′,5′-monophosphate (cGMP) accumulation in Arabidopsis roots. Exogenous application of cGMP derivative 8-bromo-cGMP increases auxin dependent primary root growth. 8-bromo-cGMP mediates this response through the degradation of AUX/IAA proteins [43]. Phosphatidylinositol-specific phospholipase C2 also governs root growth in Arabidopsis as *plc2* mutant showed defects in auxin-mediated multiple root growth phenotypes, including primary root growth [44]. An Arabidopsis exocyst complex subunit SEC6 gene regulates primary root growth through polar auxin transport and PIN protein recycling [45]. WRINKLED1 (WRI1), a key transcriptional regulator of fatty acid biosynthesis, regulates primary root growth through alteration in auxin homeostasis. The *wri1-1* loss-of-function mutants showed an increased amount of indole-3-acetic acid (IAA)-Asp conjugates and increased abundance of GH3.3, a gene involved in auxin degradation [46]. In rice, auxin influx carrier OsAUX3 regulates root development as *Osaux3-1* and *Osaux3-2* mutants showed shorter primary roots (PRs) [47]. In addition, a WUSHEL-related homeobox protein, OsWOX4, regulates primary root elongation in rice. OsWOX4 regulates this response by direct promoter binding and transcriptional activation of OsAUX1 [48]. A multidrug and toxic compound extrusion (MATE) transporter also governs root development through the modulation of auxin homeostasis in the roots [49]. In soybean, GmYUC2a is an important regulator of auxin biosynthesis during root development and nodulation [50]. Beyond transcriptional control, auxin may regulate root growth responses through other, alternate mechanisms such as protein phosphorylation. Through the impact of different auxin concentrations on the root tip, Nikonorova et al. 2021 identified global auxin-mediated changes in protein abundance and phosphorylation regulating root growth promotion and inhibition [51]. This MS-based phosphoproteome approach identified novel growth regulators, such as members of the receptor-like kinases and MAP kinases. This study also suggests that auxin, H+-ATPases, cell wall modifications, and cell wall-sensing receptor-like kinases are tightly embedded in pathways underlying cell elongation. In addition, MKK2 was found to be a potential novel regulator of root growth regulating auxin biosynthesis and signalling [51].

**Figure 2:**
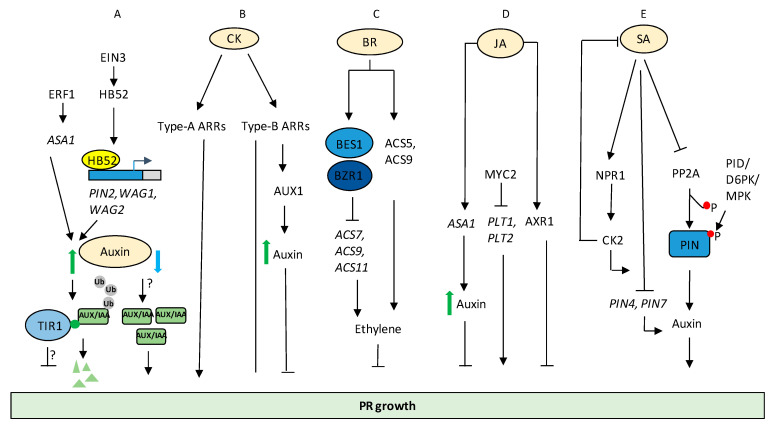
Regulation of primary root (PR) growth by various hormones and their crosstalk. (**A**) Auxin effects root growth in a concentration-dependent manner and interacts with ET signalling pathway to control PR elongation. Low concentration of auxin promotes PR growth probably via free AUX/IAAs, while high concentration inhibited PR growth through TIR1-mediated signalling via an unknown non-transcriptional mechanism (**B**) CK signalling modulates PR growth via AUX-1 mediated auxin translocation. (**C**) BR induces ET production via stabilizing ACS5, and ACS9 represses ET synthesis through BES1 and BZR1-mediated repression of *ACS7*, *ACS9*, and *ACS11* to control PR growth. (**D**) MYC2, a DNA binding bHLH transcription factor in JA signalling, represses the transcriptional expression of *PLT1* and *PLT2.* JA regulates expression of *ASA1* and modulates auxin levels to cause root growth inhibition. JA and auxinsignalling occurs via AXR1 to control PR growth. (**E**) SA via NPR1-mediated signalling establishes an auto regulatory feedback regulation between CK2 and SA to link between SA signalling and auxin transport. SA also led to inhibition of PR elongation in NPR1-independent manner via affecting PP2A, leading to changes in PIN activity and auxin export, resulting in attenuation of root growth. Solid black arrow indicates confirmed pathway. Blue arrow indicates low levels of auxin, green arrow indicates high levels of auxin. Red circle indicates phosphorylation.

Auxin regulates PR development via interaction with various other phytohormone signalling components. For example, a plant-specific and ethylene-responsive HOMEOBOX PROTEIN52 (HB52) mediates crosstalk between auxin and ethylene signalling in controlling PR elongation. HB52 works downstream to ETHYLENE-INSENSITIVE3 (EIN3) and regulates the transcription of auxin transport-related genes, including *PIN2*, *WAVY ROOT GROWTH1* (*WAG1*), and *WAG2* through binding to their promoter regions (Figure 2A) [52]. In another report, ETHYLENE RESPONSE FACTOR1 (ERF1) mediates the interaction of auxin and ethylene signalling through the transcriptional regulation of *ANTHRANILATE SYNTHASE α1* (*ASA1*) during PR elongation [53].

Numerous studies have shown how the complex interplay between CK and auxin governs stem cell architecture and meristem size during the early stages of root development. The CK transmembrane receptor ARABIDOPSIS HISTIDINE KINASE 3 (AHK3) together with type-B ARABIDOPSIS RESPONSE REGULATORS (ARR1) and ARR12 modulate auxin redistribution in the root meristem [54]. This change in auxin distribution is brought by modulating the levels of PINs through activation of the auxin repressor SHORT HYPOCOTYL 2 (SHY2)/INDOLE-3-ACETIC ACID INDUCIBLE 3 (IAA3), leading to cell differentiation [4,54]. CK was also shown to regulate the *PIN* gene expression transcriptionally through CYTOKININ RESPONSE FACTORS (CRFs) [4]. Auxin, on the other hand, mediates the degradation of SHY2, which leads to the upregulation of *PIN* genes, leading to cell division and expansion [54,55]. Thus, CKs and auxin are involved in the homeostatic feedback regulatory loop that intersects at the common regulatory factor SHY2 to control root meristem size [4,56]. Negative regulators of CK signalling, namely the type-A ARRs, also influence the patterning of root apical meristem [57]. Higher order mutants of type-A ARRs have been shown to have smaller root meristem sizes as compared to the wild type and exhibited enhanced sensitivity towards PAT inhibitor NPA. Following exogenous application of CK, the level of PIN4-GFP was significantly reduced in the root cap. The protein levels, but not the transcript levels, of auxin efflux carriers PIN1 and PIN3 were downregulated in the type-A ARR octuple mutant (*arr3*,*4*,*5*,*6*,*7*,*8*,*9*,*15*) roots [57].

External application of CK or increased CK signalling by mutants (higher order type-A ARR) shows negative regulation of PR length both in monocots and dicots [4,58,59,60,61,62,63]. The negative role of CK in root growth is also supported by CK deficient mutants, where the PR grows faster [58]. However, a study on over-expression of cytoplasmic CYTOKININ OXIDASE 7 (CKX7) showed conflicting results of reduced root developmental phenotype from that of other CKXs [64]. Type-B ARR higher order mutants of Arabidopsis displayed increased root length, suggesting a negative role of CK in PR length [65,66,67]. In contrast to this general phenomenon, a recent study showed a novel role of type-B ARRs in positively regulating PR length in rice [68]. Lowering of CK signalling through single and double mutants of CK receptors can increase root meristem size and activity [58,69]; however, CK receptor triple mutant is CK-insensitive and shows decreased root meristem size and activity [69,70,71]. This suggests that a minimum of CK signalling is required for PR development, and inhibitory levels of CK result in lowering of PR growth. CK also controls mitotic divisions in the QC directly by repressing the expression of auxin influx carriers AUXIN RESISTANT 1 (AUX1) and LIKE AUXIN RESISTANT (LAX2) in an ARR1- and ARR12- dependent manner. CK induces mitotic divisions in the QC as evident by the loss-of-function higher order type A ARRs [57]. Consequently, higher endogenous levels of CK in the CK oxidase mutants, ckx3 and ckx5, displayed higher divisions in the QC in comparison to the WT [72], further indicating the positive role of CK in QC divisions. However, the divisions were enhanced in the QC of lax2 roots similarly to CK treated plants, suggesting that CK represses LAX2 expression in the QC. The report also shows that LAX2 is a direct transcriptional target of ARR1. Additionally, the reduction of *LAX2* transcript in response to cytokinin was compromised in *ahk2ahk4* and *arr1-3arr12-1*, suggesting that cytokinin receptors and type B ARR are necessary for the regulation of *LAX2* [72]. Studies also demonstrate how auxin-CK genetic circuits are modulated by common regulatory genes. AUX1-mediated auxin translocation also regulates CK-dependent root growth inhibition and elongation at the root tip. AUX1 is activated downstream to type B ARR and leads to shootward auxin transport, thereby causing an increase in auxin activity and inhibition of cell elongation at the root tip (Figure 2B) [73]. Another report shows that a rice gene encoding NAC transcription factor 2 (OsNAC2) expressed in the PR tips, crown roots, and LR primordia, affects auxin and CK signalling genes. OsNAC2 physically interacts with the promoters of auxin inactivating GRETCHEN HAGEN (GH3.6 and GH3.8), AUXIN RESPONSE FACTOR 25 (OsARF25), and CYTOKININ OXIDASE 4 (OsCKX4), thus facilitating the integration of auxin and CK signalling to modulate root development in rice [74].

Brassinosteroid (BR) is another key player regulating root growth and development [75,76]. BR regulates root growth in a dose-dependent manner; both high and low doses negatively affect PR growth. Both BR signalling mutants as well as *bri1-ems-suppressor 1* (*bes1-D*) (gain-of-function) mutants have short roots. Additionally, external BR treatment negatively affects PR length [77,78,79]. Contradictory to these reports, in another study, low concentrations of BRs have been shown to promote root growth in WT plants as well as BR-deficient mutants [80], although the changes are small [80]. BR has been shown to affect both cell proliferation and cell elongation in a concentration-dependent manner to modulate root meristem size [81]. All these reports indicate that an optimal level of BR is crucial for root growth and meristem homeostasis. There is a complex cross-talk of BR signalling, BR catabolism, and auxin synthesis culminating in a pattern formation of BRASSINAZOLE-RESISTANT 1 (BZR1) in the meristem and elongation zone. It has been found that low levels of BZR1 are required to maintain the QC, whereas high levels are required in the elongation zone. It has also been shown that a balance between auxin signalling and BR signalling is required to maintain normal root growth [77]. Additionally, BR biosynthetic mutant *de-etiolated-2*-9 (*det2-9*)*,* having a short root phenotype, showed increased biosynthesis of ethylene and thereby high accumulation of ethylene. This short root phenotype of *det2-9* mutant was partially recovered in mutants *det2-9acs9* and *det2-9ein3eil1*, which are defective in ethylene synthesis and signalling, respectively. Exogenous BR has been shown to regulate ethylene biosynthesis in a dose-dependent manner. BR induces ethylene production via stabilizing ACC SYNTHASE 5 (ACS5) and ACC SYNTHASE 9 (ACS9) (Figure 2C). However, BR signalling represses ethylene synthesis through BES1 and BZR1 mediated repression of ACC SYNTHASE 7 (ACS7), ACS9, and ACC SYNTHASE 11 (ACS11) (Figure 2C). In addition, increased superoxide anions also contribute to the short root phenotype of *det2-9* mutants. BR regulates superoxide accumulation via the peroxidase pathway [82]. All these studies indicate that BR regulates root growth through multiple mechanisms. Similarly, BR signalling behaves differently in the epidermal and stele region. BR signalling promotes stem cell proliferation in the epidermal region; however, it induces differentiation of stem cells in the stele region. BR signalling induces target genes in the epidermis particularly related to auxin signalling, but mostly represses genes in the stele [83].

Although involved in mitigating biotic and abiotic stress responses [84,85,86], jasmonic acid (JA) and salicylic acid (SA) are also involved in regulating various parameters of root growth and development. The inhibitory effect of JA on plant growth was one of the first physiological responses reported in the 1980s [87,88]. The first mutant insensitive to JA-mediated inhibition of root growth was *jasmonate resistant 1* (*jar1*), which was later cloned and characterised as JA-Ile synthase [89]. Apart from this, various components of JA signalling also participate in this physiological response. A screen for *Arabidopsis* mutants unresponsive to root growth inhibition by a bacterial toxin and the JA homologue, coronatine (COR), revealed *coronatine insensitive 1* (*coi1-1*), a null mutant insensitive to JAs [90]. COI1 is at the interface of JA and ethylene (ET) signalling in mediating root growth inhibition. Previous reports show that *coi1-16* shows unresponsiveness to ET induced root growth inhibition in light, but not in dark. Furthermore, this response did not require any other components of JA biosynthesis and signalling such as *jar1-1*, *jasmonate insensitive 1* (*jin1*), *allene oxide synthase* (*aos*), or *oxophytodienoate-reductase3* (*opr3*). Thus, the inhibition of Arabidopsis root growth to 1-aminocyclopropane-1-carboxylate (ACC) is light, *COI1* dependent, but JA independent and occurs due to inhibition in cell elongation [91,92]. MYC2, a DNA binding bHLH transcription factor in JA signalling, acts as an activator of JA-induced root growth inhibition [93]. Previous reports suggest that MYC2 directly represses the transcriptional expression of *PLT1* and *PLT2* (Figure 2D) [94]. Moreover, JA may increase auxin levels by inducing the expression of auxin biosynthetic gene *ASA1*, thereby leading to root growth inhibition (Figure 2D) [95]. Another example of potential interaction between JA and auxin signalling occurs via AUXIN RESISTANT 1 (AXR1). In addition to its role in auxin signalling, *axr1-1* shows JA-mediated root growth inhibition and pathogen susceptibility (Figure 2D) [96]. Taken together, the above reports propose that JA signalling is linked to auxin homeostasis, leading to root growth inhibition. An interesting report claims that both endogenous and exogenous JA have an important role in governing the root architecture of *Helianthus annuus* seedlings. Micromolar concentrations of JA reduced the PR and LR lengths along with LR number by reducing the cortex cell length and cell divisions. However, when JA biosynthesis inhibitor Ibuprofen (IBU) was applied, an increase in cell elongation was observed, thus suggesting that endogenous JA also affect root phenotype in *Helianthus annuus* [97]. Interestingly, auxins were shown to be not essential for JA induced root growth inhibition in sunflower [97]. Studies have shown that the auxin-JA resistant double mutant (*jar1-1axr1-3*) was more resistant than the single mutants to inhibition of root growth by methyl jasmonate (MeJA). In addition, the level of resistance between single mutant *axr1-3* and double mutant *jar1-1axr1-3* was not different when IAA was applied. Additionally, JA produced its phenotype even in the presence of an auxin transport inhibitor. Besides, auxin produced its phenotype even when ibuprofen was applied. Thus, JA appears to act through two pathways: one linked to auxin, and a second via an auxin independent pathway [97]. A recent report highlights the synergistic role of JA and auxin in stem cell activation and root regeneration. Wound induced JA causes ETHYLENE RESPONSE FACTOR 109 (ERF109) activation, which further stimulates *CYCLIN D6*; 1(*CYCD6;1*) and *ETHYLENE RESPONSE FACTOR 115* (*ERF115*). They then modulate RETINOBLASTOMA-RELATED (RBR)-SCARECROW (SCR) module to allow root tissue regeneration. Upon wounding, auxin accumulation also takes place, which then activates several regeneration regulators of this pathway [98].

SA, in a concentration-dependent manner, regulates root growth and development through interaction with the auxin machinery. A report by Zhao and coworkers (2015) suggests that exogenous application of 50 µM of SA promoted root waving along with a reduction in PR elongation in Arabidopsis. This SA-mediated root waving response was regulated via *NITRIC OXIDE-ASSOCIATED PROTEIN1* (*AtNOA1*) and *NONEXPRESSER OF PATHOGENESIS-RELATED GENES 1* (NPR1). *Atnoa1* mutants displayed a disrupted root waving phenotype as a result of altered auxin distribution at the root tip, suggesting interplay of SA signalling and auxin transporters through *AtNOA1* to modulate root waving in Arabidopsis [99]. Another report shows that exogenous application of 100 µM SA inhibited root elongation in Arabidopsis [100]. Armengot et al. 2014 revealed that the mutants of protein kinase CK2, a Ser/Thr kinase accumulated high levels of SA, which further resulted in the impediment of PR growth (Figure 2E). *CONSTITUTIVE PATHOGEN RESPONSE* (*CPR*) mutants having high constitutive levels of SA exhibited a similar phenotype as the CK2 loss-of-function mutants. In addition, SA enhanced the transcription of CK2 encoding genes via the NPR1-mediated signalling, thereby implying an auto regulatory feedback regulation between CK2 and SA [101]. Loss of CK2 activity led to reduction in the SA-mediated repression of auxin transporter genes including *PIN4* and *PIN7,* thus establishing a link between SA signalling and auxin transport [101]. Exogenous application of 3 µM SA inhibits PR elongation and causes shortening of cell length. SA led to auxin redistribution and a pronounced auxin response at the root tip in a *PATHOGENESIS–RELATED 1* (*PR1*)-independent manner. At concentrations higher than 50 µM SA, the growth related processes in the root were ceased. Lower concentrations of SA promoted the expression of *TRP AMINOTRANSFERASE OF ARABIDOPSIS 1*(*TAA1*) and *PIN1* and suppressed *PIN2* and *PIN7* expression. A high concentration of SA upregulated the expression of *TAA1* and inhibited the expression of PIN efflux transporters [100]. Utilizing mathematical modelling of auxin distribution after SA exposure, Pasternak et al. 2019 hypothesized that root tip accumulated auxin after treatment with low concentration of SA, whereas high concentrations of SA led to depletion in auxin levels. Accumulation of auxin after the application of a lower level of SA causes the distal meristem to become enlarged. Endodermal periclinal divisions were higher in Arabidopsis root treated with a lower concentration of SA as a result of enhanced auxin levels in the endodermis. However, lesser vascular cell files were demonstrated in SA-treated roots in comparison to control due to reduced accumulation of auxin in the vascular cells [100]. However, administration of 1.5 µM SA under stress-free conditions increased the total root biomass in *Zea mays* [102]. SA-deficient mutant *abnormal inflorescence meristem1* (*aim1*) in *Oryza sativa* exhibits a defect in root growth and showed reduced meristem activity. Reduced SA levels in *aim1* led to further decrement in the reactive oxygen species (ROS) levels due to enhanced expression of ROS scavenging-related genes. The authors hypothesize that *AIM1* is involved in SA biosynthesis and represses the ROS scavenging genes to accumulate ROS for higher root meristem activity via induction of transcriptional repressors WRKY62 and WRKY76 [103]. Recent observations by Tan et al. 2020 concluded that SA led to inhibition of PR elongation in an NPR1-independent manner (Figure 2E). The reduced PR growth was a result of inhibition of serine/threonine PROTEIN PHOSPHATASE 2A (PP2A) activity by SA, leading to increased phosphorylation of PIN protein by different kinases such as PINOID (PID)/D6PK/MAPKs, thereby affecting PIN activity andauxin export, resulting in attenuation of root growth (Figure 2E) [104].

### 3.2. Lateral Roots

**Figure 3:**
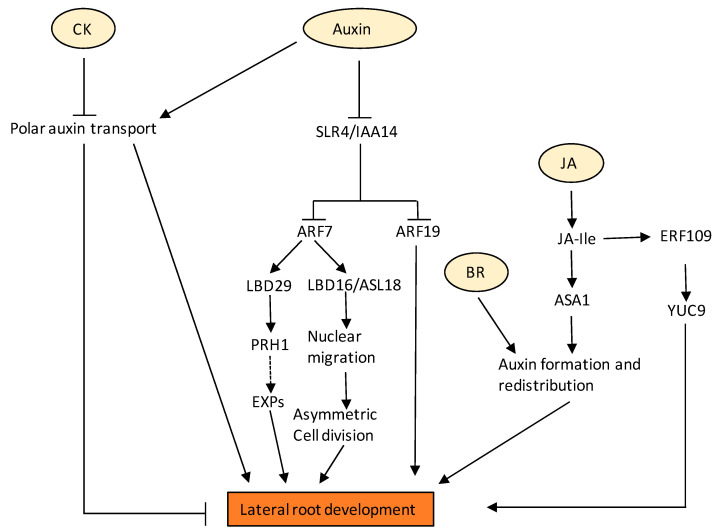
Regulation of LR development by various hormones and their crosstalk. Auxin via SLR4/ARF7-ARF9 signalling module promotes LR development. Auxin-induced expression of *PRH1* is dependent on ARF7 and LBD29. PRH1 may promote LR development by regulating the expression of *EXP* genes that promote cell wall loosening. SLR4/ARF7-ARF9 signalling module also activates the expression of LBD16/ASL18 that are involved in the symmetry breaking of LR founder cells for LR initiation and primordium development. JA positively regulates lateral root formation by modulating auxin biosynthesis and homeostasis. JA induces the expression of *ASA1* both through COI1 and ERF109. ERF109 also induces the expression of another auxin biosynthetic gene, *YUC9*. BR regulates lateral root development by interacting with auxin. At low concentration, BR modulates auxin transport and promotes lateral root development. CK negatively regulates LR initiation and development by inhibiting auxin carriers. Solid black arrows indicate confirmed pathway. Dotted arrow indicates pathways for which there is little or no evidence.

Auxin is commonly known to induce LR formation as suggested in different auxin biosynthesis and signalling mutants in Arabidopsis and tomato [105,106]; however, there are reports suggesting that exogenous auxin application (25nM) reduced the induction of LRs [107]. In the roots, a regular pattern of root branching depends on the oscillatory gene activity that determines prebranch sites. A recent report suggests that local auxin source in the root cap is derived from the auxin precursor indole-3-butyric acid (IBA), which regulates the oscillation amplitude and therefore determines the origin of the prebranch site. An IBA-regulated gene, *MEMBRANE-ASSOCIATED KINASE REGULATOR4* (*MAKR4*), converts the prebranch site into a regular spacing of lateral organs [108]. LBD16/ASYMMETRIC LEAVES2-LIKE18 (ASL18), which is a direct downstream target of SOLITARY ROOT (SLR)/IAA14-ARF7-ARF19 auxin signalling module, regulates LR development possibly through transcriptional regulation through its related LBD/ASLs, causing nuclear migration and asymmetric cell division for LR initiation and primordium development (Figure 3) [109]. A very recent report shows the role of ABCB transporter in LR emergence [110]. The report suggests that loss of *ABCB21* reduces rootward auxin transport and delays LR emergence [110]. These results support a primary role for ABCB21 in regulating auxin distribution supplementary to the primary ABCB auxin transporters ABCB1 and 19 [110]. It is a well-established fact that ARF7 and the LATERAL ORGAN BOUNDARIES DOMAIN (LBD) module are required for Arabidopsis LR development. A recent report suggests that PR1 homolog *PRH1* regulates LR development in Arabidopsis via the auxin signalling pathway. It was shown that the expression of *PRH1* was induced upon auxin, and mutants of *PRH1* had fewer LRs. Additionally, *PRH1* was shown to be a direct transcriptional target of ARF7 and LBDs [111]. PRH1 might then regulate the expression of EXPANSIN (EXP) genes, which then affect cell wall loosening (Figure 3).

Although the optimization of LR development is primarily governed by auxin, various studies highlight the role of CKs in LR patterning and growth [60,63,112]. Previous reports demonstrate that change in the CK levels, either through increased endogenous signalling or through external application, inhibits LR production [113,114]. In line with this, a decrease in CK signalling increased LRs [58,65,71,115]. CK insensitive mutants have increased LR formation as a result of increased auxin perception [114], whereas LR formation in *ahk* triple mutant was severely impaired as a result of a decrease in cell division [70]. The inhibition of LR by CK is brought indirectly by changing auxin transport by disrupting PIN1 localization (Figure 3) [116]. This results in a change of cell division pattern of LRFC in the outer layer from undergoing periclinal division [113]. Upon application of exogenous CK, a rapid modulation in PIN1 dynamics on the plasma membrane showed the role of CK in controlling auxin flow. CK also led to the post-transcriptional degradation of PIN1 proteins through targeted lysis in vacuoles [116]. The endocytic trafficking of PIN proteins is regulated by AHK4 and type-B ARR (ARR2 and ARR12) [117]. Plants perturbed in their perception and/or signalling of CKs have large meristem sizes [71] and a higher number of LRs [65], and exhibit increased root branching phenotype [58], implying the important role of CKs in maintaining homeostasis in regulating RSA [66]. A recent report claims that the impact of CK on LR elongation is tissue- and concentration-specific as low CK levels reduced the length of the laterals in the apical/proximal region of the root system. By contrast, high levels of CK reduced the length along the entire root system, but with a more pronounced effect in the apical zones [63]. Another interesting report introduces TRANSPORTER OF IBA1 (TOB1), as a new player in LR development. Michniewicz and co-workers show that the *TOB1* gene is responsible for transport of IBA to the cytoplasm and converts it into active IAA. They also show that it is a direct target of the CK response pathway, and mutants altered in *TOB1* show an increased number of emerged laterals. Thus, *TOB1* acts as a link between CK signalling and auxin to fine-tune RSA [118].

Apart from their role in inhibiting LR growth, CKs regulate the spacing of LRP along the PR in Arabidopsis, as disruption of CK signalling genes led to irregularities in spacing of consecutive LRP sites [119]. High local endogenous CK levels in pericycle cells neighbouring the pre-existing LRs inhibit LRP initiation at proximity to existing ones. This spacing is regulated via blockage of pericycle founder cells from G2 to M phase transition by CK [60,119,120].

BRs are also one of the major factors regulating LR development. BRs modulate LR development primarily via interacting with auxin. BR signalling mutant *brassinosteroid insensitive 1* (*bri1*) has been reported to have a decreased number of LRs, suggesting the possible involvement of BRs in LR development. Furthermore, a low concentration of BR has a positive effect on LR numbers. BRs promote the initiation of LR primordia by increasing acropetal auxin transport (Figure 3) [121]. At low levels of auxin, BRs and auxin exhibited a synergistic effect on LR formation, which was inhibited by NPA, an auxin transport inhibitor [121]. However, higher concentrations of BRs have a negative effect on LR formation [122]. Higher concentrations of BRs have been shown to induce the expression of several AUX/IAAs, which play a vital role in LR formation [123]. These reports suggest that high concentrations of BRs inhibit LR formation, possibly via inhibiting auxin signalling through these AUX/IAAs [81]. CK has been shown to antagonise the effect of BRs on LR development. CK receptor double mutants *ahk2ahk3* showed increased sensitivity to BRs in LR elongation, indicating the antagonistic actions of these two hormones in LR development [113].

Various reports strongly suggest the role of JA in LR development. It was found that the promoters of all four members of *ALLENE OXIDECYCLASE* (*AOC*), a JA biosynthesis gene, were highly expressed in LR primordia, suggesting the involvement of JA in LR formation and development [124]. Several reports show the positive effect of JA on LR initiation and growth by affecting auxin biosynthesis and homeostasis. It was found that exogenous application of MeJA represses LRP initiation in *asa1-1*. Transcriptional studies further validated that MeJA induces the expression of *ASA1* in a COI1-dependent manner that ultimately leads to increased local auxin accumulation in the root basal meristem and, as a result, promotes LR formation (Figure 3) [95]. Another mechanism by which JA promotes LR formation is by inducing the expression of *ETHYLENE RESPONSE FACTOR 109* (*ERF 109*), which then binds to the GCC boxes in the promoters of *ASA1* and *YUCCA 9* (*YUC9*) (Figure 3) [125]. COI1 is also involved in JA-induced pericycle cell activation and LR formation, positioning, and emergence on bends and requires a canonical auxin signalling pathway [126]. A very recent report highlights the negative role of JA in regulating auxin induced LR formation, but independently of COIreceptor [127]. Higher concentrations of (−) JA and (+) JA were shown to counter the promontory effect of auxin by stabilizing DII-VENUS and suppressing the expression of *PUCHI* and *LATERAL ORGAN BOUNDARIES-DOMAIN 29* (*LBD29*), involved in LR formation [127].

SA has been shown to enhance root biomass and a number of emerged LRs in Catharanthus roseus [128]. The distance between the root tip and the first emerged LR primordia was also decreased, which could be due to SA-induced reduction in the level of CK in the root cap or lower CK sensitivity in apical cells of the root. Femtomolar (fM) concentrations of SA displayed a larger root cap with a higher number of columella cells [128].SA also enhanced the LR formation in a dose-dependent manner [100]. Armengot and co-workers revealed that the mutants of CK2, a Ser/Thr kinase, accumulated high levels of SA, which further resulted in the impediment of LR growth [101]. Pasternak et al. 2019 reported inhibition of LR number with increasing concentrations of SA [100]. Similarly, the application of 20 µM SA or 40 µM SA to Arabidopsis seedlings inhibited LR organogenesis by changing the auxin homeostasis through the cellular redistribution of PIN proteins [104].

### 3.3. Adventitious Roots

**Figure 4:**
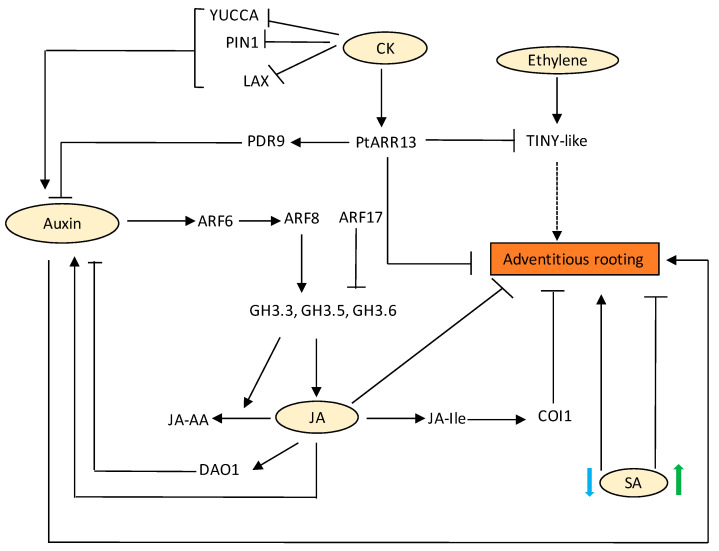
Regulation of AR development by various hormones and their crosstalk. Auxin controls AR initiation by activating ARF6 and ARF8, leading to downregulation of COI1-mediated JA signalling. GH3.3, GH3.5, and GH3.6 are regulated by ARF6, ARF8, and ARF17. The 3 GH3s control JA homeostasis. JA level controls JA-Ile levels. JA-Ile negatively regulates AR development by activating COI1-dependent signalling. Feedback regulation by DAO1 is activated by JA signalling, which then regulates IAA homeostasis. SA at low concentration promotes AR development, whereas at higher concentrations it inhibits AR development. CK regulates auxin homeostasis by negatively affecting auxin carriers *PIN*, *LAX,* and *YUCCA1* genes, thereby regulating adventitious rooting through these pathways. In poplar cuttings, CK signalling activates *PtARR13,* which represses AR formation by promoting the expression of *PDR9* and inhibiting the expression of TINY-Like TF. Solid black arrows indicate confirmed pathway. Dotted arrow indicates pathways for which there is little or no evidence. Blue arrow indicates low level of SA and green arrow indicates high level of SA.

Lateral and adventitious roots mostly share common regulatory networks except for a few regulatory mechanisms that distinguish lateral and AR pathways. AUXIN RESPONSE FACTOR 7 (ARF7) and AUXIN RESPONSE FACTOR 19(ARF19) are key molecular players in the regulation of LR initiation and transcriptionally activate *LATERAL ORGAN BOUNDARIES-DOMAIN* (*LBD*) genes. In the case of ARs, molecular functions of ARFs are overtaken by WUSCHEL RELATED HOMEOBOX 11 (WOX11) transcription factor. WOX11 responds to wound-induced auxin in the procambium and its surrounding cells and works redundantly with its homologous protein WUSCHEL RELATED HOMEOBOX 12 (WOX12) to activate downstream LBD16 and 29, resulting in the fate transition from procambium to root founder cell [129]. However, a few reports suggest that ARF7/19 also govern the formation of adventitious roots from hypocotyls, which precludes the discrimination of molecular pathways of LR and adventitious roots [130]. Auxin transporters are also important for AR development. For example, an ATP-binding cassette efflux family, ABCB19, has been linked to AR formation [131]. Other members of the same transporter are found to transport IBA [132,133], which has been tested to have the highest ability to induce adventitious rooting [134]. Other hormones such as ethylene have been shown to regulate the positioning of AR primordia [135]. In the fern, *Ceratopteris richardii*, an intermediate clade WOX (IC-WOX) gene, *CrWOXA*, which is exclusively expressed in root founder cells during LR and AR initiation, regulates AR organogenesis. CrWOXA is a direct target of the auxin signalling pathway, which in turn may directly activate the WUSCHEL-clade WOX (WC-WOX) gene, CrWUL [136]. A similar case is observed in Arabidopsis, where *WOX5/7* expression is directly regulated by *WOX11/12* required for root founder cell division during AR initiation [137]. AR initiation is mediated by ARF6, 8, and 17 modules, which control the expression of *GH3.3*, *GH3.5*, and *GH3.6* (Figure 4). ARF 6 and 8 are positive regulators, whereas ARF17 is a negative regulator, thus altering auxin homeostasis. Thus, a complex regulatory network exists that fine-tunes AR initiation (Figure 4) [138,139].

Research conducted on crop plants indicates the role of CK in concert with auxin in the regulation of AR induction and development. In apple rootstocks, N6-benzyladenine (BA) was shown to inhibit AR formation via restricting root primordium growth [140]. In tomato, zeatin levels increased during AR induction and extension, which was reduced after IAA treatment [141]. CK also negatively affect PIN, YUCCA6 and LAX3 expression, leading to reduced auxin flow and hence, AR repression (Figure 4) [135]. Transcriptomic studies dealing with poplar softwood cuttings revealed that *PtARRs* were significantly downregulated during a time course of 6 h that was concomitant with an increase in auxin signalling genes, depicting a general antagonism between auxin and CK during AR induction [142]. The *PtARR13* (a positive CK regulator) overexpressing shoots exhibited differential expression of two most important genes; *PLEIOTROPIC DRUG RESISTANCE TRANSPORTER9* (*PDR9*) and *TINY*, which are induced via auxin and ethylene, respectively, in normal WT excised shoots [142], implying that CK inhibits AR development through interactions with other hormonal networks (Figure 4). In *Arabidopsis*, the CK receptor *ahk4* mutants, also known as *wooden leg-3* (*wol-3*), and the higher order mutant *ahk2ahk3ahk4* exhibited enhanced AR formation on the hypocotyl [70,143]. Phenotypic analysis of plants overexpressing the CK-degrading enzymes such as the *CYTOKININ OXIDASE/DEHYDROGENASE* (*AtCKX*) gene family also demonstrates the role of CKs in AR formation. The *35S:AtCKX2* and *35S:AtCKX4* lines displayed enhanced AR, which was almost 7 times as much in comparison to the WT [58]. However, in *Vigna* callus, kinetin alone was able to induce rhizogenic activity of the callus in terms of AR formation [144] pointing out the fact that distinct signals in different plant species determine specific outcomes in terms of AR induction and formation. BR has been reported to induce adventitious rooting in cucumber at lower concentrations through a nitric oxide-dependent pathway, whereas a higher concentration was inhibitory [145].

JA differentially regulates AR formation in different species. In Arabidopsis, JA negatively regulates adventitious rooting via COI1 and MYC2/3/4 dependent JA signalling pathway by altering the JA-Ile homeostasis. *jar1-1*, *coi1-16*, *myc2*, *myc3,* and *myc4* form numerous adventitious roots as compared to the WT, and this response was dependent on ARF 6 and ARF8 (Figure 4) [139]. Additionally, GH3.3, GH 3.5, and GH3.6 catalyse the production of inactive forms of JA, such as JA-Asp, JA-Trp, and JA-Met, thus controlling JA homeostasis and AR initiation (Figure 4) [139]. However, JA enhanced the AR formation in leafy cuttings of *Petunia hybrid* [146]. Very recently, Druege and co-workers established that early wound-induced JA accumulation leads to enhanced IAA accumulation and hence stimulates AR production in Petunia cuttings [147]. The effect of JA in AR development depends on experimental conditions. At low sub-micromolar concentrations, MeJA has been shown to promote AR development in dark grown hypocotyls of Arabidopsis and thin cell layers of *N. tabacum* when applied together with IBA and kinetin [148]. Recently Lakehal and co-workers proposed that TIR1 and AUXIN SIGNALING F-BOX 2 (AFB2) interact with IAA6, IAA9, and IAA17 to control JA homeostasis and AR initiation in Arabidopsis [138]. They also showed that a feedback circuit exists between IAA and JA that is mediated by DIOXYGENASE FOR AUXIN OXIDATION (DAO1) and COI1-dependent JA signalling. DAO1 catalyses the conversion of IAA to oxindole-3-acetic acid (oxIAA), hence leading to reduced free IAA levels and AR initiation in Arabidopsis. They also showed that the expression of DAO1 is induced by JA signalling (Figure 4) [149].

SA has also been shown to promote the formation of secondary adventitious roots in Arabidopsis seedlings at a lower concentration (30 µM) and showed inhibition of AR growth at concentrations higher than 30 µM SA [100]. At a concentration of 200 µM, SA initiated AR formation in mung bean (*Phaseolus radiatus* L.) hypocotyl cuttings, with maximum effect at 400 µM SA. Further increases in SA concentration inhibited the response. Treatment of mung bean hypocotyl cuttings with N, N’-dimethyl thiourea (DMTU), an H_2_O_2_ scavenger, led to a decline in the number of ARs. Similarly, diphenyleneiodonium (DPI), a specific inhibitor of membrane-linked NADPH oxidase, also restricted AR formation, suggesting H_2_O_2_ functions synergistically with SA to induce adventitious rooting [150].

### 3.4. Root Hair

**Figure 5:**
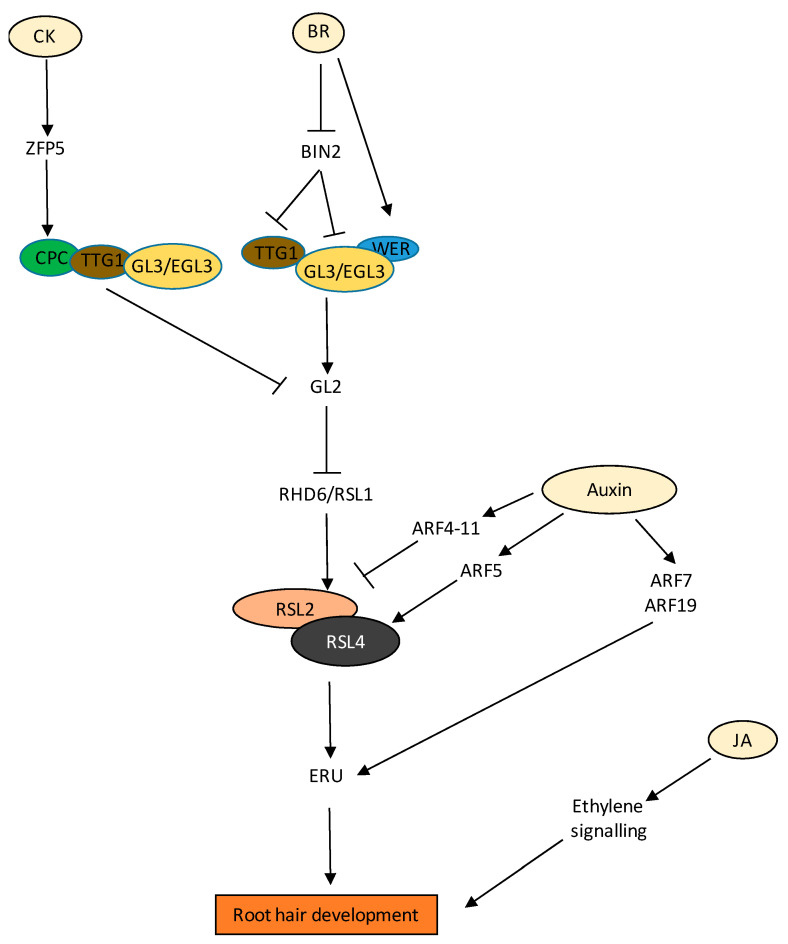
Regulation of RH development by various hormones and their crosstalk.BR positively regulates the expression of WER, GL2 (negative regulator of RH development) and inhibits BIN2, which is then unable to phosphorylate GL3/EGL3 and TTG1. Presence of BR promotes functional TF complex formation between TTG1, GL3/EGL3, and WER, promoting *GL2* expression, leading to inhibition of the RH development pathway. CK induces TF ZFP5, which promotes the expression of CPC (positive regulator of RH development), leading to the formation of functional TF complex of TTG1, GL3/EGL3, and CPC. The complex inhibits the expression of GL2, thus causing activation of the RH development pathway. The presence of auxin liberates the ARFs from AUX/IAAs. The ARFs show different specificity to RH development. ARF4-11 inhibits, whereas ARF6 promotes, the RH development pathway. ARF5 directly interacts with the promotor of *RSL4*, leading to RH development. Auxin also directly regulates the expression of RSL4 target gene ERU via ARF7/ARF19. JA positively influences RH growth via ET signalling. Solid black arrows indicate confirmed pathways.

Phytohormone auxin promotes the growth of RH [151]. ARF5/7/8/19 bind to the promoter of *RSL4* to control its expression in trichoblasts [152]. ARF19 was also shown to regulate the expression of *RSL2*(Figure 5) [153]; however, overexpression of auxin repressors ARFs in RH (*ARF1-4*, *ARF9-11*, and *ARF16*) suppressed RH growth (Figure 5) [154], suggesting differential activity of ARFs. Among other phytohormones, it was found that auxin and ethylene act synergistically in regulating RH growth. A transcriptome study suggests that almost 90% of the auxin-regulated genes underlying RH growth were synergistic to ethylene [25]. Moreover, other hormones such as JA, BR, and strigolactone pathways may regulate root hair growth possibly through interconnections with the auxin/ethylene signalling pathways [155]. BR inhibits root hair growth. The expression of root hair-related AUX/IAA (such as AXR2, AXR3, and SLR) was increased by the application of EpiBL and repressed in the BR-insensitive *bri1* mutant, suggesting a possibility that BR may suppress root hair growth through the suppression of auxin signalling. JA positively regulates root hair growth. The interconnection of JA and auxin was shown as axr2 mutants were found to be resistant to exogenous JA in primary root inhibition, and JA was also shown to promote auxin biosynthesis. However, no direct evidence supports idea that JA regulates root hairs through auxin (extensively reviewed by Lee and Cho, 2013). In RH cells, auxin promotes membrane depolarization in a concentration- and pH-dependent manner that is strongly dependent on AUX1-mediated auxin transport. AUX1-mediated auxin transport involved SCF TIR1/AFB-type Ca^2+^ signalling in the RH as treatment with SCFTIR1/AFB inhibitor auxinole blocked AUX1 transport and IAA-triggered calcium signals [156]. Using 3D modelling, Jones et al. 2009 showed that AUX1-dependent auxin transport via non-hair cells maintains the auxin flow in the development of RH [157]. In a recent study, auxin was found to govern the transcriptional regulation of a cell wall receptor protein ERULUS (ERU) in an ARF7- and 19- dependent manner (Figure 5). ERU is a member of the *Catharanthus roseus* RECEPTOR-LIKE KINASE 1-LIKE (CrRLK1L) subfamily of cell wall receptors and regulates RH cell wall composition and modulates pectin dynamics [26]. MEDIATOR18 (MED18) protein, through the modulation of auxin signalling and transport, influences the growth of PR, LR, and RH development [158]. The energy status of plants also affects RH development. In a recent study, glucose-activated TOR was found to phosphorylate and stabilize PIN2 at the plasma membrane, which results in the shootward auxin transport to the RH [159,160].

There are very few reports citing the development of RH by CK. CK promotes RH formation at the differentiation zone regulating a similar set of RH-specific genes, as observed for auxin and ethylene (Zhang et al. 2016). CK independently regulate RH formation to that of auxin and ethylene (Zhang et al. 2016). CK levels are also modulated in RH of root nodule-forming species, such as in *Medicago trancatula* via several cytokinin biosynthesis and signalling genes, including *CYTOKININ RESPONSE 1*(*CRE1*) and type A ARRs such as *MtARRA2*, *MtARRA8*, *MtARRA9*, and *MtARRA10* in response to nodulation factors, indicating that CK signalling negatively impacts root epidermal infections [161,162].

In Arabidopsis, 6-Benzylaminopurine (BAP) treated seedlings showed enhanced RH growth in response to low hormonal concentrations (10 µM) [163]. Apparently, this profound effect was auxin- and ethylene-independent, as similar results were achieved in *axr1*, *ethylene response 1* (*etr1*), and ethylene inhibitor Aminoethoxyvinylglycine (AVG) treated seedlings, respectively (Zhang et al. 2016), while on the other hand, *CKX2* overexpression line (*35S:CKX2*) displayed abnormal short-hair phenotypes [161]. In addition, several proteins and TFs regulate RH development, and are themselves orchestrated by upstream phytohormonal networks. This is exemplified via molecular characterization of the C_2_H_2_ zinc finger protein, ZINC FINGER PROTEIN 5 (ZFP5), whose mutant has fewer RHs than the WT [164]; its expression is transcriptionally increased by BAP application (Figure 5). In effect, ZFP5 itself induces the expression of another RH-localised RB MYB protein, CAPRICE (CPC), involved in RH patterning by binding to its promoter, which then inhibits negative regulators of RH development such as GLABRA2 (GL2) (Figure 5) [165].

In the case of RH, the relative spatial distribution of BRI1 plays a crucial role. Earlier, it was reported that BRs play an essential role in the establishment of epidermal cell fate in Arabidopsis roots. BRs convey the positional information of hair and non-hair cells along the roots via BRI1 [166]. Later, it was found that expression of BRI1 in hair cells has a promontory effect, whereas in the case of non-hair cells, its expression is inhibitory [167]. Additionally, BRASSINOSTEROID-INSENSITIVE 2 (BIN2) also plays a key role in regulating the distribution of hair (H) and non-hair (N) cells in the root epidermis. Root hair formation is determined by transcription factor complex formation and lateral communication between H- and N-cells. At high BR concentrations, BIN2 regulates this distribution primarily via the phosphorylation of two major transcription factors, ENHANCER OF GLABRA3 (EGL3) and TRANSPARENT TESTA GLABRA 1 (TTG1), leading to functional TF complex and subsequent *GL2* expression and determination of the non-hair cell fate in all epidermal cells (Figure 5) [168]. It has also been reported that expression of BRI1 under protophloem-specific promoters rescued root meristem phenotypic defects of *bri1 brl1 brl3* triple receptor mutants [169]. All of these results suggest that not only optimal concentration of BR but tissue-specific expression of BR signalling components also play a crucial role in root development.

JA and MeJA also positively influence RH growth via ethylene signalling (Figure 5) [170]. It was found that the effects of JAs were abolished in the ethylene-insensitive mutants *etr1-1* and *etr1-3*, or by ethylene biosynthesis inhibitors (AVG) [170].

### 3.5. Gravitropism

**Figure 6:**
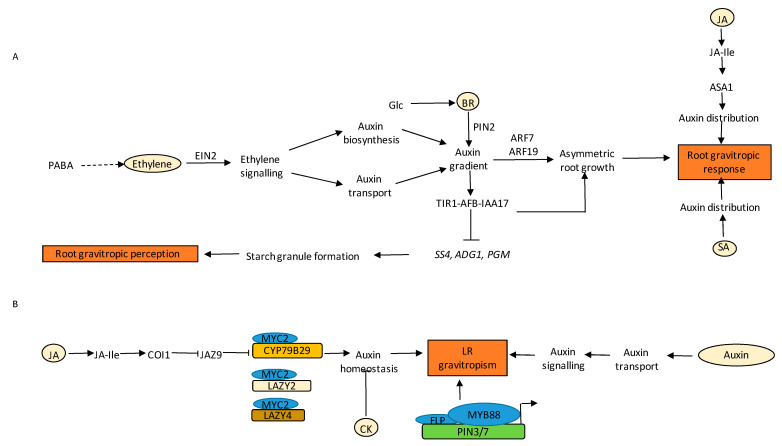
Regulation of PR and LR gravitropism by various hormones and their crosstalk. (**A**) PABA works upstream of ET signalling, through which it regulates auxin biosynthesis and transport. This ultimately leads to ARF7/19 mediated auxin activity effects on asymmetric growth promotion followed by root gravitropic response. Glc acts via BR to regulate root gravitropism. BR regulates root gravitropism by interacting with auxin via PIN2 distribution. Auxin signalling module TIR1-AFB-IAA17 mediates starch granule formation and gravitropic perception by inhibiting the expression of *PGM*, *ADG1*, and *SS4*. JA influences gravitropic response by modulating auxin levels through SA1 induction. SA affects auxin transport and redistribution, leading to gravitropic bending. (**B**) The presence of JA activates COI1-mediated JA signalling that causes proteasomal degradation of JAZ9, leading to the liberation of MYC2, which then binds to the promotors of *CYP79B2* and *LAZ2* and *LAZY4*, leading to changes in auxin homeostasis and LR gravitropism. Auxin transport and signalling modules are essential to regulate vertical orientation of LRs. The binding of FLP and MYB88 TFs leads to transcriptional activation of PIN3 and PIN7, which then lead to gravitropic bending of LRs. CK perturbs auxin homeostasis, leading to horizontal branching of LRs. Solid black arrows indicate confirmed pathways.

Auxin is the major hormone that drives the growth of an organ (PRs or LRs) towards the gravity vector. The angle at which an organ grows with respect to the gravity vector is called the gravitropic set point angle (GSA) [171]. It is a well-established fact that the distribution of auxin drives the root growth in response to gravity via the starch statolith hypothesis [172]. In response to gravity-stimulation of PRs, polarized localization of PIN3 and PIN7 redirects auxin flux towards the lower side of the PR, thus, causing differential cell elongation and root tip bending [173,174,175]. The dual role of auxin in gravity perception and gravity response was demonstrated by Zhang and co-workers. They reported thatTIR1-dependent auxin signalling module TIR1-AFB-IAA17 mediates starch granule formation and gravitropic perception in root tips by regulating the expression of starch granule synthesis genes *PHOSPHOGLUCOMUTASE* (*PGM*), *ADENOSINE DIPHOSPHATE GLUCOSE PYROPHOSPHORYLASE* (*ADG1*), and *STARCH SYNTHASE 4* (*SS4*) (Figure 6A) [31]. Downstream signalling of auxin reorients PR growth. Auxin influx and efflux transporters distribute differential auxin gradient in the upper and lower sides of the gravity-stimulated roots. The asymmetric distribution of auxin towards the lower side of the root is modulated by AUX1 and PINs [175,176]. While auxin is the central component of root gravitropic responses, ET is implicated in this process through crosstalk with auxin, which also involves *para*-aminobenzoic acid (PABA) (Figure 6A) [176]. The interference of ET in root graviresponses is mediated through its interaction with the auxin pathway at the level of auxin biosynthesis and transport. In gravistimulated roots, PABA promotes an asymmetric auxin response, which causes the asymmetric growth responsible for root curvature. This activity requires the auxin response transcription factors AUXIN RESPONSE FACTOR7 (ARF7) and ARF19 as well as ethylene biosynthesis and signalling, indicating that PABA activity requires both auxin and ethylene pathways (Figure 6A) [176].

Biochemical and computational analyses have revealed that besides auxin, CK also influences gravity responses in plants [112,177,178]. The combinatorial effect of auxin-CK on root gravitropic responses is observed at the protein transport and differential expression levels of PINs [178]. Several mutant lines defective in endogenous CK perception and signalling such as *ahk3* and higher-order mutants; *ahk2 ahk3 ahk4*, *ahp1 ahp2 ahp3 ahp4 ahp5*, *arr1 arr10 arr12,* and the CK degrading lines *35S:AtCKX2* and *35S:AtCKX3*, had higher root gravitropic angles than the WT [178]. The expression of *PIN3* was also shown to be significantly lower in these lines as compared to the WT in the root tip plasma membrane of columella cells. However lateral expansion of PIN7 levels was also observed in the columella cells, although the intensity was comparable to the WT [178]. It is known that CK alters auxin sensitivity and transport in root columella cells. AUX1 signals were diminished in *35S:AtCKX2* and *35S:AtCKX3* lines, suggesting endogenous CKs regulate AUX1 in root tips [178]. Root caps cells were also shown for their potential in producing CKs upon gravistimulation in Arabidopsis, indicating the importance of de novo CK synthesis [179,180]. The *ARR5* exhibited an asymmetrical pattern and redistribution at the lower side following a 90 degree shift in root orientation [180]. The tips aligned towards BAP-applied sites, causing a discernible bending and triggering an inhibition in root elongation during gravity sensing [180].

There are several reports indicating the involvement of BR signalling in the regulation of root gravitropic responses. Exogenous BR application is known to increase gravitropic curvature of PRs [181,182]. BR signalling mutants showed reduced gravitropic curvature, whereas transgenic plants overexpressing BRI1 were hypersensitive to gravity. BR and auxin show a synergistic effect on gravitropic curvature at low concentrations of auxin primarily via increasing acropetal and basipetal auxin transport [181,182]. BR has also been found to promote the activity of RHO-RELATED PROTEIN FROM PLANTS 2 (ROP2) GTPase, resulting in enhanced polar accumulation of PIN2 protein and thereby increased gravitropic response [182]. However, high concentrations of auxin antagonise BR-induced root gravitropic curvature and vice versa [183]. Exogenous BR could also affect gravitropic responses by regulating the actin cytoskeleton and PIN2 localization pattern, and thereby the auxin gradient in a way similar to auxin [184]. A recent report indicated that BR antagonises PIN2 endocytosis [185]. BR-mediated modulation of endocytic sorting and distribution of PIN2 plays a crucial role in the formation of lateral PIN2 gradient in gravistimulated roots. This lateral gradient regulates auxin signalling in gravistimulated roots and thereby root growth deviation (Figure 6A) [185]. In addition to this, BR plays a key role in glucose (Glc) mediated root directional responses (Figure 6A) [186]. Exogenous BR dramatically increased Glc-mediated directional responses. Additionally, BR signalling mutant *bri1-6* showed reduced root deviation in the presence of Glc, whereas *bzr1-1D* showed increased root deviation in thepresence of Glc. Glc enhances BR signalling by increasing the endocytosis of BRI1. It was found that BR modulates Glc-induced root deviation responses by regulating PAT (Figure 6A) [186]. A recent report has implicated the involvement of BR signalling in root gravitropic response in maize. Treatment with BR resulted in more curvature of PRs of maize [187].

JA is also found to be a crucial regulator of root gravitropism. Studies on rice coleoptiles revealed an increased amount of JA upon gravistimulation. In addition, a JA gradient was formed opposite to the internal auxin gradient across the stimulated organ during a gravitropic response that worked in an IAA manner. Consistent with these observations, a JA-deficient rice mutant, *hebiba*, bent slowly upon gravitropic stimulus, thus, suggesting that JA might accelerate the bending response [188]. Moreover, Trp conjugates of JA (JA-Trp) that act as IAA antagonists have been reported to cause agravitropism in a dose-dependent fashion. The response is TIR1-dependent but COI1-independent [189]. JA also influences gravitropism via ASA1, which further leads to changes in auxin homeostasis (Figure 6A). Thus, JA might work via auxin to regulate tropic responses in roots. Another report demonstrates that MeJA impairs lateral auxin distribution in WT roots upon gravistimulation and therefore slows root gravitropic response [190].

Administration of SA led to inhibition of gravitropic response in maize roots, which was an indirect result of inhibition of ethylene biosynthesis by SA. In the recent studies by Tan and co-workers, the effect of exogenous SA on various root growth parameters was examined. They concluded from their studies that exposure of 20 µM or 40 µM SA to Arabidopsis seedlings led to partial agravitropic roots in an NPR1-independent manner [104]. SA also affected the auxin redistribution in the PRs and was involved in the gravitropic bending via auxin efflux carrier PIN2. SA leads to the agravitropic response via blocking PP2A activity, causing the cellular SA levels to increase, thereby resulting in phosphorylation of PIN proteins and affecting auxin transport and redistribution in the cell (Figure 6A) [104]. SA also modulates root gravitropism by affecting clathrin-mediated endocytosis, as exposure to 50 µM SA in wild type Arabidopsis seedlings led to a reduction in gravitropic root curvature. However, the *clathrin heavy chain* (*chc2-2*) exhibited less sensitivity to SA-induced gravitropic response [191]. A recent study by Ke et al. 2021 reported that high concentrations of SA impaired the root gravitropism in Arabidopsis seedlings via condensation of PIN2 into hyperclusters through REMORIN 1.2 (REM1.2) dependent nanodomain compartmentalization. As a result, the movement of PIN2 by clathrin-mediated endocytosis and lateral diffusion was restricted, thereby interfering with the asymmetric distribution of auxin, leading to the inhibition of gravitropic responses [192].

Not only the main root, but other parts of the RSA such as LRs also show gravitropism [193,194]. In such cases, the LRs partially suppress positive ortho-gravitropic growth and grow radially to cover more area in the soil. Supplying exogenous auxin and an endogenous increase in auxin levels causes LRs to grow at a more vertical GSA. Reports by Roychoudhry and co-workers have shown that the entire TIR1/AFB-Aux/IAA-ARF-dependent auxin signalling module is necessary to establish GSA in LRs (Figure 6B) [194]. Recently emerged LRs (stage I) show strong but transient *PIN3* expression, presumably limiting the strength of auxin redistribution in (stage II) LRs that are establishing their GSA [193,195]. At later stages, the expression of *PIN3* diminishes, and *PIN4* and *PIN7* are expressed to maintain vertical GSA [193]. While auxin promotes gravitropic bending of LRs, CK function as an anti-gravitropic component that is shown to suppress cellular elongation in the upper portion of stage II LRs, thus promoting the radial expansion of RSA (Figure 6B) [112]. Apart from this, there are other molecular components, such as *LAZY* gene family in Arabidopsis, that has also been reported to have a role in maintaining GSA by controlling the distribution of PINs. *LAZY* (*LZY*)*/DEEPER ROOTING* (*DRO*)*/NEGATIVE GRAVITROPIC RESPONSE OF ROOTS* (*NGR*) genes affect auxin flow towards the lower side of the LR in gravistimulated LRs. The *lzy1lzy2lzy3* mutant showed an inverse auxin distribution and asymmetry in the *PIN3* expression in the columella cells of the LR, resulting in a horizontally placed LR [196,197]. A recent report by Sharma et al. 2020 also showed a promising involvement of JA-auxin machinery in the regulation of LR angle via *LAZY* genes. JA signalling transcription factor MYC2 directly activates the transcription of *CYP79B2*, an auxin biosynthetic gene, and *LAZY2/4* in the regulation of LR angle. In addition, JA treatment also affects auxin transport machinery and PIN2 localization in the stage II LR, leading to a downward LR orientation (Figure 6B) [198]. The Arabidopsis R2R3-MYB transcription factor FOUR LIPS (FLP) and its paralog, MYB88, act redundantly but differently in the regulation of primary and LR gravity responses. FLP alone is responsible for the transcription regulation of *PIN3* and *PIN7* in gravity-stimulating cells in the PRs, whereas FLP-MYB88 both act redundantly to control LR gravitropic set point angle (GSA) (Figure 6B) [5]. The Arabidopsis FERONIA (FER) receptor kinase, which integrates multiple hormonal and environmental signals, governs auxin-mediated LR and root gravitropic responses. The *fer-4* mutants showed increased LR branching and delayed gravitropism, which was found to be associated with aberrant PIN2 polarity and an altered polar auxin transport [199]. Previous reports revealed that NGR is necessary for the positive gravitropism of roots. A recent study suggests that NGR, a plasma membrane protein exclusively expressed in the columella and LR cap cells, governs positive gravitropism through regulation of auxin efflux carrier PIN3 localization and modulation of lateral auxin flow in response to gravity [200]. A *Lotus japonica* mutant defective in *LAZY3*, a functional ortholog of *LAZY1*, showed negative gravitropism in primary and LRs. This negative gravitropic response in *lazy3* mutant was due to reduced polar auxin transport [201]. In Arabidopsis, *DEEPER ROOTING 1* (*DRO1*) mutation causes horizontally placed LRs and altered GSA, while *DRO1* overexpression showed steeper LR angles [202]. In a recent report, *Atdro1* primary and LRs showed defects in establishing an auxin gradient in response to gravity [203]. Collectively, all of these results indicate a promising involvement of active auxin signalling and transport machinery in the regulation of root gravitropic responses in LRs.

## 4. Conclusions and Future Prospects

The RSA is continuously modified by abiotic and biotic signals from the soil that interact with various endogenous cues and turn them into different cellular responses. Phytohormones affect several overlapping processes and their actions depend on specific hormone combinations rather than on the action of a single hormone individually. This review has focussed on various examples of crosstalk between auxin-JA, auxin-CK, etc., suggesting that different hormones affect a number of common responses in plants [95,96]. Moreover, these interactions do not occur via a simple linear mechanism; rather, there are many feedback loops and the involvement of various downstream activators and repressors that drive a single development output robustly. For example, binding of MYC2 transcription factor on the promoters of *LAZY2*, *LAZY4*, and auxin-responsive gene *CYP79B2* leads to gravitropic bending of LRs [198]. A recent review by Semeradova et al. 2020 summarizes that auxin transport machinery is post-translationally modified by multiple hormones that enable the rapid modulation of the auxin flow to control plant growth and development [204]. Additionally, several other reports as mentioned in this review have further clarified how almost all plant hormones influence the rate of auxin homeostasis [95,118,149], thus making it clear that auxin is indispensable in controlling all aspects of the plant life cycle.

It is very essential to map key transcriptional and post translational pathways and their genes that define the distinct nature of PR and LRs, as that might be the key in understanding hormonal and developmental outputs. Another interesting area is to pinpoint DNA polymorphisms that control a particular root trait of interest. This provides a powerful approach to identify new root branching regulatory genes. A challenging step will be to study the regulation of different root traits under more realistic conditions than under highly aseptic growth conditions such as agar plates where we might miss novel adaptive strategies employed by the plants to survive in their microenvironment. Thus, there is a need to integrate systems biology-aided computational methods (SimRoot) [205] and computer technology such as magnetic resonance imaging and microscale computed tomography [206] that will not only enable us to integrate information related to the overall organization of how roots sense various endogenous and environmental signals, and how they turn them into cellular responses, but also assist in devising predictive models that may identify the crucial regulators, hubs, and integrators involved in regulating RSA. Such multiscale mechanistic insights will underpin efforts to develop crops with improved root systems.
